# Transcriptome Analysis of the Anti-TGFβ Effect of *Schisandra chinensis* Fruit Extract and Schisandrin B in A7r5 Vascular Smooth Muscle Cells

**DOI:** 10.3390/life11020163

**Published:** 2021-02-20

**Authors:** Sanghoon Lee, Jung Nyeo Chun, Hae-Jeung Lee, Hyun Ho Park, Insuk So, Ju-Hong Jeon, Eun-Jung Park

**Affiliations:** 1 Department of Physiology and Biomedical Sciences, Seoul National University College of Medicine, Seoul 03080, Korea; Sanghoon.lee.moon@gmail.com (S.L.); jungnyu@snu.ac.kr (J.N.C.); insuk@snu.ac.kr (I.S.); 2 Institute of Human-Environment Interface Biology, Seoul National University, Seoul 03080, Korea; 3 Department of Food and Nutrition, College of BioNano Technology, Gachon University, Gyeonggi-do 13120, Korea; skysea@gachon.ac.kr; 4 College of Pharmacy, Chung-Ang University, Seoul 06974, Korea; xrayleox@cau.ac.kr

**Keywords:** bioinformatics, gene expression profiling, master regulator analysis, *Schisandra chinensis*, vascular smooth muscle cell

## Abstract

*Schisandra chinensis* fruit extract (SCE) has been used as a traditional medicine for treating vascular diseases. However, little is known about how SCE and schisandrin B (SchB) affect transcriptional output-a crucial factor for shaping the fibrotic responses of the transforming growth factor β (TGFβ) signaling pathways in in vascular smooth muscle cells (VSMC). In this study, to assess the pharmacological effect of SCE and SchB on TGFβ-induced transcriptional output, we performed DNA microarray experiments in A7r5 VSMCs. We found that TGFβ induced distinctive changes in the gene expression profile and that these changes were considerably reversed by SCE and SchB. Gene Set Enrichment Analysis (GSEA) with Hallmark signature suggested that SCE or SchB inhibits a range of fibrosis-associated biological processes, including inflammation, cell proliferation and migration. With our VSMC-specific transcriptional interactome network, master regulator analysis identified crucial transcription factors that regulate the expression of SCE- and SchB-effective genes (i.e., TGFβ-reactive genes whose expression are reversed by SCE and SchB). Our results provide novel perspective and insight into understanding the pharmacological action of SCE and SchB at the transcriptome level and will support further investigations to develop multitargeted strategies for the treatment of vascular fibrosis.

## 1. Introduction

Fibrosis is a characteristic pathological feature of vascular diseases, such as atherosclerosis and restenosis [1,2,3]. Transforming growth factor β (TGFβ) has a profound pro-fibrotic effect on vascular tissues [3,4] by affecting a wide range of biological pathways, including cell proliferation and migration, inflammation, and trans-differentiation as well as the accumulation of extracellular matrix (ECM) proteins [1,5,6,7]. Particularly, TGFβ induces the synthetic, non-contractile phenotypes (e.g., cell proliferation and migration) of vascular smooth muscle cells (VSMCs) in response to vascular injury [8,9]. Therefore, the TGFβ signaling pathway has gained attention as a plausible target for attenuating vascular fibrosis [10,11].

TGFβ signals through the type II receptor kinase (TβRII)-mediated activation of TβRI [12,13]. The activated TβRI propagates its downstream signaling through both the Smad-dependent canonical pathways and the Smad-independent non-canonical pathways. In the canonical pathways, TβRI phosphorylates Smad2 and Smad3 to form a heteromeric complex with Smad4. The Smad complex enters the nucleus to initiate the transcription of its target genes [14,15]. In the non-canonical pathway, TβRI stimulates non-Smad signaling pathways, including extracellular signal-regulated kinase (ERK), phosphoinositide 3-kinases (PI3K), Rho-associated coiled-coil kinase (ROCK), and inhibitor of nuclear factor-κB kinase (IKK), which in turn, activate a range of transcription factors [16,17]. These results indicate that the transcriptional and phenotypical output of TGFβ signaling is determined by the cooperation between Smad- and non-Smad signaling cascades determines the transcriptional of TGFβ signaling, which leads to fibrotic changes.

**Figure 1:**
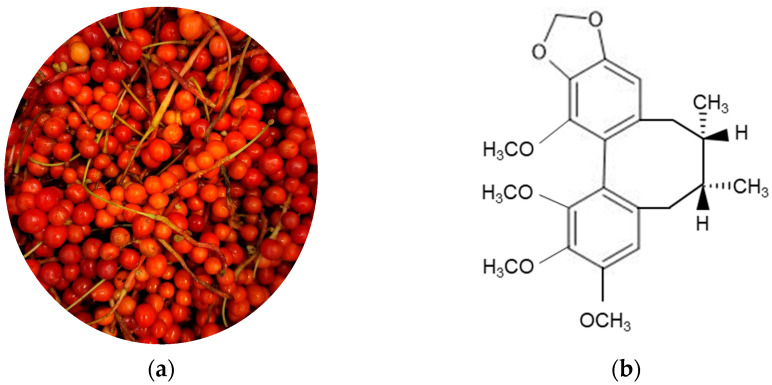
*Schisandra chinensis* fruit and structure of schisandrin B. (**a**) *Schisandra chinensis* fruit; (**b**) schisandrin B.

*Schisandra chinensis* fruit (Figure 1a) as an oriental herbal medicine has been used traditionally to treat various human diseases [18]. Recently, accumulating evidence suggests that *Schisandra chinensis* fruit and its active ingredients have a potential role in the treatment of vascular fibrosis [19]. We have reported that *Schisandra chinensis* fruit extract (SCE) and its ingredient schisandrin B (SchB, Figure 1b) have a potent anti-fibrotic activity by suppressing the TGFβ signaling pathways in VSMCs [20,21,22]. SCE and SchB block TGFβ-induced phosphorylation and nuclear translocation of the Smad complex, which decreases the expression of ECM proteins [20]. In addition, SCE and SchB inhibit the phosphorylation of myosin light chain in a Smad-independent manner, which leads to suppressing in actin stress fiber formation and cell migration [21]. Moreover, SCE and SchB attenuate TGFβ-mediated activation of IKKα/β, thereby inhibiting NF-κB activity [22]. However, little is known about how SCE and SchB affect the transcriptional output of the TGFβ signaling pathways. 

In this study, we performed DNA microarray experiments in A7r5 VSMCs to examine the mechanism of action of SCE and SchB at the transcriptomic level. We found that TGFβ induced transcriptome changes in VSMCs and that these changes were significantly reversed by SCE and SchB. Further computational genome-wide analysis provided a global picture of the pharmacological effect of SCE and SchB on TGFβ-mediated biological processes and fibrotic changes. Our results provide novel perspective and insight into future translational research and into the development of healthcare strategies.

## 2. Materials and Methods

### 2.1. DNA Microarray Experiments and Data Processing

DNA microarray experiments were performed using total RNA from A7r5 cells, following treatment with 100 mg/mL SCE or 10 μM SchB for 24 h in the presence or absence of 1 ng/mL TGFβ1 (R&D Systems) as described in our previous papers [22,23]. SCE were prepared by ethanol extraction using ultrasonic bath and SchB was purified from SCE using the HPLC system as described previously [20,24]. The microarray data are available through the Gene Expression Omnibus (GEO) database under the accession number GSE87439. The raw microarray data were normalized using single-channel array normalization (SCAN) method [25]. SCAN is a Bioconductor R package, and R version 3.6.0 was used for all analyses. Microarray probe sets were mapped to 14,065 genes using a custom mapping file, Rat2302_Rn_ENTREZG (version 23.0.0), which is provided by the BrainArray resource [26].

### 2.2. Collection of Public Microarray Data 

To construct A7r5 VSMC-specific transcriptional interactome, public microarray datasets were collected from the GEO database (GSE15713 and GSE21573) and normalized using the SCAN method. These data were combined with our microarray data (GSE87439), which are available from GEO database (GSE134932). The batch effect among the data sets was removed as described in our previous papers [27,28,29].

### 2.3. Cluster Validation and Differentially Expressed Gene (DEG) Selection

Our microarray data consist of six experimental groups depending on the reagents used in the experiments: group 1 (vehicle), group 2 (SCE alone), group 3 (SchB alone), group 4 (TGFβ alone), group 5 (TGFβ + SCE), and group 6 (TGFβ + SchB). Feature selection across six groups was performed using the Linear Models for Microarray Data (Limma) Bioconductor R package with a multiclass statistical problem type [30]. The Benjamini and Hochberg (BH) procedure was used for the adaptive control of the false discovery rate (FDR) in multiple testing [31]. The significant features were determined by the threshold FDR q-value 0.2. Internal clusters were validated by hierarchical clustering and principal component analysis (PCA) using the selected features.

### 2.4. Functional Assessment for DEGs

For deep functional assessment of the enriched gene signatures, the DEGs identified by Limma (less strict threshold FDR q-value 0.3) across six groups were applied to Gene Signature Enrichment Analysis (GSEA) with Hallmark gene signatures (version 6.2 at Molecular Signatures Database (MSigDB), http://software.broadinstitute.org/gsea (accessed on 10 February 2021)) in a pairwise way (i.e., TGFβ versus vehicle, SCE versus vehicle, SchB versus vehicle, TGFβ + SCE versus TGFβ, and TGFβ + SchB versus TGFβ). Significantly enriched gene signatures were determined by the threshold FDR q-value 0.2.

### 2.5. Protein-Protein Interaction Network Analysis

From the functional assessment of DEGs, we identified SCE- and SchB-effective genes (details in Section 3). We used Search Tool for the Retrieval of Interacting Genes/Proteins (STRING) database (ver. 11.0) [32] to perform protein-protein interaction analysis for the common genes between SCE- and SchB-effective. We set required interaction score 0 and edge color for interaction evidence.

### 2.6. Master Regulator Analysis (MRA) Using A7r5 Cell-Specific Transcriptional Interactome 

Algorithm for the Reconstruction of Gene Regulatory Networks (ARACNe) was used to construct an A7r5 cell-specific transcriptional interactome as described in our previous papers [28,29]. The Rattus norvegicus transcription factors (TF) were collected from Animal Transcription Factor Database 3.0 (AnimalTFDB 3.0). From the combined microarray data (GSE134932), a consensus gene network was generated by 100 rounds of ARACNe bootstrapping (http://califano.c2b2.columbia.edu/aracne/ (accessed on 10 February 2021)). MRA-Fisher’s exact test (FET) was used to infer master regulator candidates and their transcriptional targets in A7r5 cell-specific transcriptional interactome. The ARACNe preprocessing and MRA-FET analysis were run in geWorkbench software version 2.6.0 (http://wiki.c2b2.columbia.edu/workbench/i-ndex.php/Home (accessed on 10 February 2021)).

## 3. Results

### 3.1. Distinctive Changes in the Gene Expression Profile in A7r5 Cells

**Figure 2:**
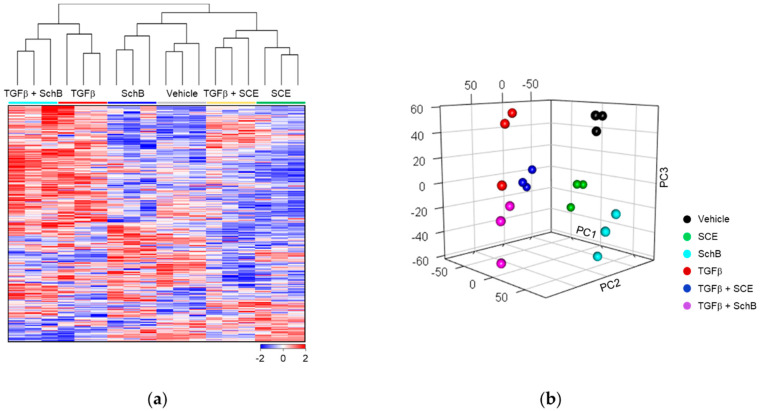
Distinctive gene expression profile in A7r5 cells. (**a**) Hierarchical clustering analysis illustrates the global gene expression differences among the six experimental groups. (**b**) Principal Component Analysis (PCA) shows distinctive clusters in the principal components. In the 3-dimentional PCA plot, the first three principal components were used to present the samples.

TGFβ stimulates the transcription of a range of its target genes through both Smad-dependent and -independent pathways [15,17]. To better understand the pharmacological effect of SCE and SchB on vascular fibrosis, we performed DNA microarray experiments in A7r5 cells. Through feature selection, we identified 9549 DEGs across six experimental groups. Hierarchical clustering analysis and PCA demonstrated that these six groups were clustered into discrete ones (Figure 2a,b). These results indicate that each group shows distinctive changes in the gene expression profile.

### 3.2. SCE and SchB Reverse TGFβ-Induced Changes in the Gene Expression Profile

**Figure 3:**
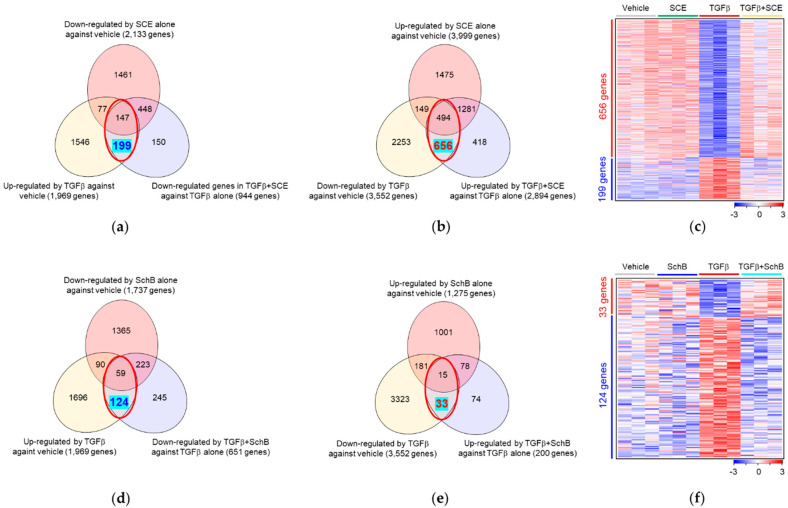
Identification of SCE- and SchB-effective genes. The numbers of SCE- (**a**–**c**) and SchB-effective genes (**d**,**e**) are presented as red or blue in Venn diagrams (**a**,**b**,**d**,**e**) and heatmaps (**c**,**f**).

To identify DEGs among experimental groups, we performed Limma analysis in a pairwise way and presented the results as Venn diagrams (Figure 3a,b,d,e) or heatmaps (Figure 3c,f). We found 5521 DEGs (1969 up-regulated and 3552 down-regulated) in TGFβ against vehicle (Tables S1 and S2) and we defined these genes TGFβ-reactive genes. Additionally, we found 3838 DEGs (2894 up-regulated and 944 down-regulated) in TGFβ + SCE against TGFβ (Tables S3 and S4), and 851 DEGs (200 up-regulated and 651 down-regulated) in TGFβ + SchB against TGFβ (Tables S5 and S6). On the other hand, we found 6132 DEGs (3999 up-regulated and 2133 down-regulated) in SCE versus vehicle (Tables S7 and S8) and 3012 DEGs (1275 up-regulated and 1737 down-regulated) in SchB versus vehicle (Tables S9 and S10). 

To assess the pharmacologic action of SCE and SchB at the transcriptome level, we identified the TGFβ-reactive genes but reversed by SCE. We counted the DEGs up-regulated by TGFβ, but down-regulated by TGFβ+SCE or vice versa (red circles, inter-sections between two bottom Venn diagrams in Figure 3a,b). Then, we excluded the DEGs by SCE solely (top Venn diagrams in Figure 3a,b) to cancel the effect of SCE irrespective of TGFβ. Therefore, we found that SCE reversed the expression of 855 out of 5521 TGFβ-reactive genes (199 out of 1969 up-regulated genes and 656 out of 3552 down-regulated genes, highlighted in light blue in Figure 3a–c, Table S11). We defined these genes SCE-effective genes. Likewise, SchB reversed the expression of 157 out of 5521 TGFβ-reactive genes (124 out of 1969 up-regulated genes and 33 out of 3552 down-regulated genes, highlighted in light blue in Figure 3d,e, Table S12). We defined these genes SchB-effective genes. The number of SCE-effective genes, 855 (199 + 656) is much larger than the number of SchB-effective genes, 157 (124 + 33). These results indicate that SCE has a broader pharmacologic effect than its active component SchB in terms of the TGFβ-induced transcriptome.

**Figure 4:**
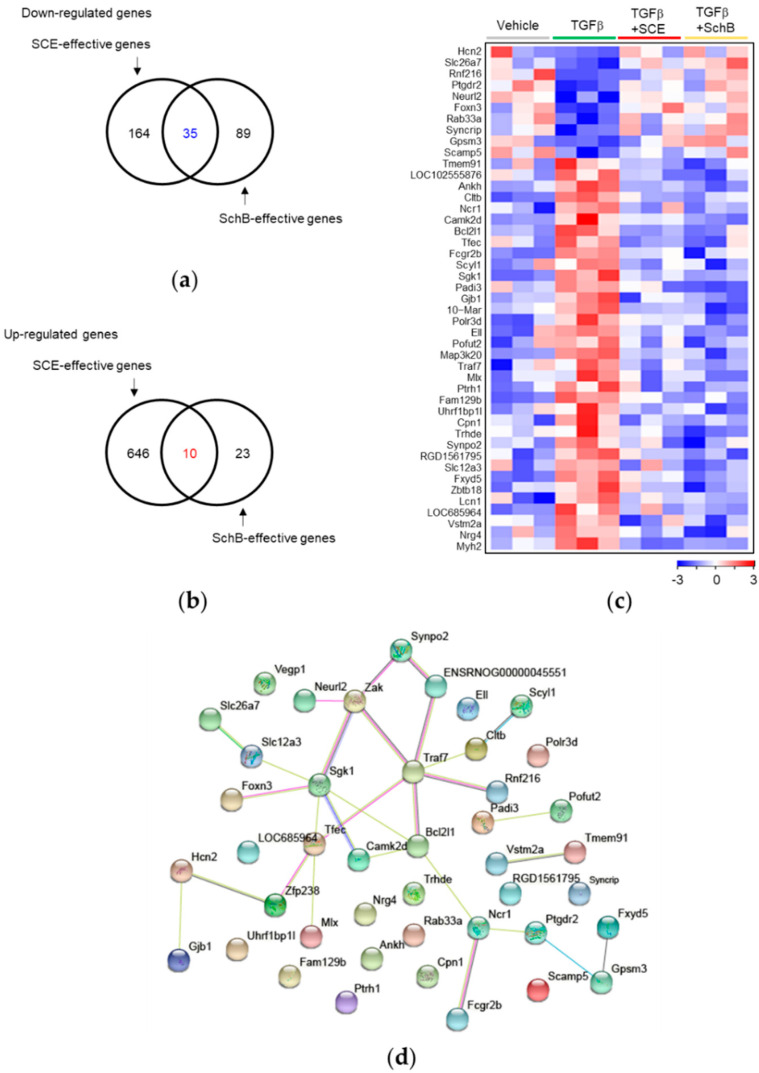
The common DEGs between SCE- and SchB-effective genes. Venn diagrams show commonly down-regulated (**a**) or up-regulated (**b**) by TGFβ + SCE or TGFβ + SchB versus TGFβ. (**c**) Heatmap representation of the common 45 DEGs. (**d**) Protein-protein interaction on the common DEGs between SCE- and SchB-effective genes.

Among SCE- and SchB-effective genes (i.e., TGFβ-reactive genes whose expression levels are reversed by SCE and SchB), 10 genes are commonly up-regulated, and 35 genes are down-regulated by TGFβ + SCE or TGFβ + SchB versus TGFβ (Figure 4a–c). Among these 45 genes, SGK1, and CAMK2D have been known to play crucial roles in vascular fibrosis [33,34]. In addition, many other genes, such as NRG4, SCYLI, MYH2, FXYD5, NCR1, LCN1, and FCGR2B, have been reported to be associated with fibrosis in various tissues, including liver, heart, and lung [35,36,37,38,39,40,41]. In protein–protein interaction network analysis on the common DEGs between SCE- and SchB-effective genes, a total of 29 connections (edges) were identified among 45 proteins (nodes) with average node degree of 1.35 (Figure 4d). Gene Ontology cellular component identified that the identified nodes are involved in vesicle coat- and plasma membrane-associated functions with FDR *q*-value < 0.05. These results suggest that our computational analysis can be useful for discovering novel therapeutic targets or predictive markers for TGFβ-mediated vascular fibrosis.

### 3.3. Functional Assessment of the Biological Effect of SCE and SchB by GSEA 

**Figure 5:**
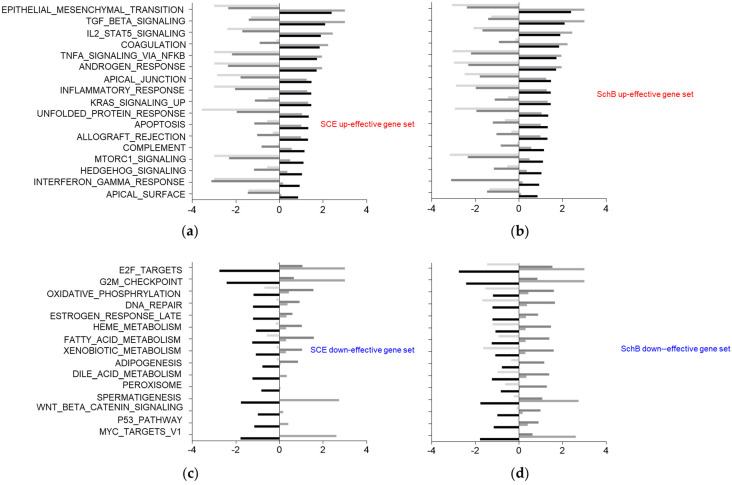
The gene signatures regulated by SCE- or SchB-effective genes. SCE (**a**,**c**) and SchB (**b**,**d**) reverse TGFβ-induced up-regulated (**a**,**b**) or down-regulated (**c**,**d**) Hallmark gene signatures. Positive normalized enrichment score (NES) means up-regulation in TGFβ versus vehicle, and negative NES means down-regulation in TGFβ + SCE or TGFβ + SchB versus TGFβ.

To assess the molecular signatures and biological effects of SCE and SchB on TGFβ-induced phenotypic changes, we performed GSEA using Hallmark gene signatures on the 9549 DEGs that were identified through feature selection. We found that SCE and SchB reversed TGFβ-induced up-regulated Hallmark gene signatures, such as EPITHELIAL_MESENCHYMAL_TRANSITION, TGF_BETA_SIGNALLING, TNFA_SIGNALING_VIA_NFKB, and MTORC1_SIGNALING signatures, but that TGFβ-induced down-regulated Hallmark signatures, such as E2F_TARGETS, G2M_CHECKPOINT, and FATTY_ACID_METABOLISM signatures, compared to vehicle (Figure 5a–d, Tables S13 and S14). The Hallmark signature ‘TGF_BETA_SIGNALLING’, as an internal control, was readily reversed by SCE or SchB (Table S13). These results demonstrate that SCE and SchB potently suppress the changes in a range of TGFβ-induced biological processes, which can contribute to their anti-fibrotic activity.

### 3.4. Master Regulators (MRs) That Regulate SCE- and SchB-Effective Genes

**Figure 6:**
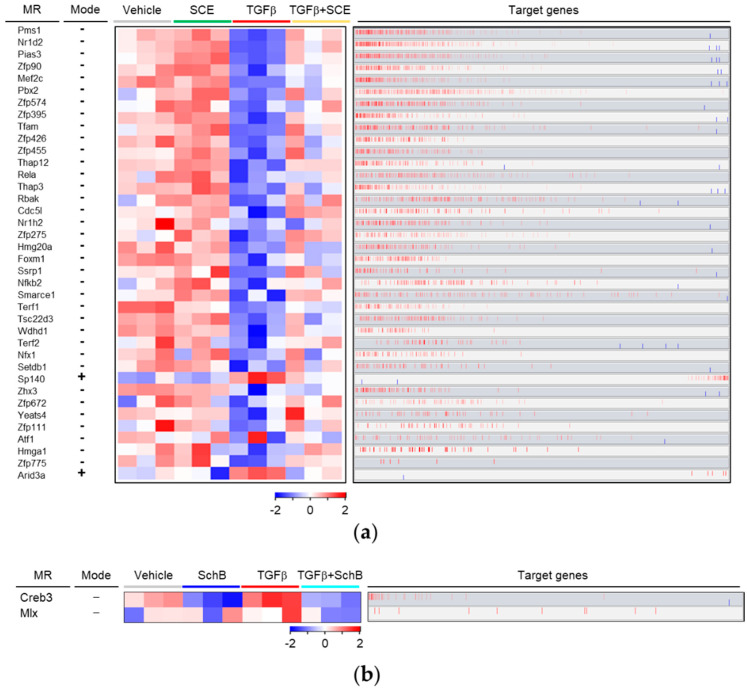
Identification of MRs that regulate the expression of SCE- or SchB-effective genes. MRA predicted 38 or 2 TFs as MRs that control the expression of SCE- (**a**) or SchB-effective genes (**b**), respectively. The heatmap shows the differential gene expression of the TFs across four groups. The mode indicates up-regulation (+) or down-regulation (−) of the individual MRs in TGFβ + SCE or TGFβ + SchB versus TGFβ. The bar graph denotes the positive (red) or negative (blue) correlation between individual MRs and their target genes (Spearman’s correlation between the expression levels of the MR and its targets).

For a mechanistic understanding of the transcriptional regulation by SCE and SchB, we constructed an A7r5-specific transcriptional interactome using ARACNe algorithm and performed MRA. ARACNe inferred a small consensus network of 17,645 interactions among the 891 Rattus norvegicus TF hub markers (genes) and the 14,004 genes. From this transcriptional interactome, the MRA predicted 38 or 2 TFs as MR candidates that regulate the expression of SCE- or SchB-effective genes, respectively (Figure 6a,b, Table 1 and Table 2).

Among these MR candidates, RELA Proto-Oncogene (RelA) is the most extensively studied molecule in fibrosis, including vascular fibrosis. Particularly, RelA plays an important role in aldosterone- or TGFβ-mediated fibrotic changes in VSMCs [22,33]. Our MRA found 31 putative target genes of RelA (Table S15), providing a clue to future investigation for the molecular mechanisms underlying RelA-mediated fibrotic responses. In addition, our literature review confirmed that other genes, including Mef2c, Pias3, Tfam, NFκB2, Terf2, Nr1h2, and Foxm1, have also been reported to be associated with fibrosis of various organs, such as heart, liver, lung, kidney, and mammary gland, rather than fibrosis of vascular tissues [42,43,44,45,46,47,48,49,50,51,52,53]. These results illuminate the validity of our computational analysis results, suggesting that our results will contribute to discovering novel mechanism of fibrotic changes and novel pharmacological actions of SCE and SchB.

## 4. Discussion

We have reported the effect of SCE and its ingredient SchB on the Smad-dependent and -independent TGFβ signaling cascades in VSMCs [20,21,22]. However, little is known about their effect on TGFβ-induced transcriptional output, which is crucial for shaping fibrotic responses. In this study, our computational analysis demonstrated that SCE and SchB considerably reverse TGFβ-induced changes in terms of transcriptional output. In addition, we aggregated the public microarray data obtained from the experiments using VSMCs to identify TFs that act as MRs to regulate SCE- and SchB-effective genes. Therefore, the current paper will provide the basis of future research for understanding the pharmacologic actions of SCE and SchB in terms of gene expression regulation.

It has been known that inflammation and VSMC proliferation and migration are crucial features for vascular fibrosis [2,3]. Our computational analysis indicates that SCE and SchB attenuates inflammation processes (e.g., TNFA_SIGNALING_VIA_NFKB, IL2_STAT5_SIGNALING, INFLAMMATORY_RESPONSE, INTERFERON_GAMMA_RESPONSE) and cell migration and proliferation processes (e.g., EPITHELIAL_MESENCHYMAL_TRANSITION, MTORC1_SIGNALING, KRAS_SIGNALING) (Figure 5). In fact, SCE and SchB has been known to inhibit the inflammatory responses and synthetic phenotypes of VSMCs [19], confirming the usefulness of our transcriptomic approach. These results suggest that our work can contribute to developing predictive markers of efficacy of anti-TGFβ or anti-fibrosis therapies. In addition, our analysis raised a new possibility that SCE and SchB regulate the unfolded protein response and hedgehog signaling, which can pave a way to understand their novel pharmacological actions (Figure 5).

Our bioinformatic analysis found that SCE and SchB partially reversed TGFβ-induced changes in the gene expression profile. On the other hand, we have confirmed that SCE and SchB almost completely inhibited TGFβ-induced Smad phosphorylation and reporter gene activity [20,21]. These results suggest that SCE and SchB primarily reverse the Smad-dependent transcriptional program in TGFβ-treated cells, and partly affect the non-canonical pathway-dependent transcriptional program. Therefore, our results provide a crucial clue for dissecting specific signaling pathways that are affected by SCE and SchB.

In traditional medicine, the crude extracts of plants have been preferentially used over their isolated ingredients. Because the crude extracts consist of various active ingredients and these ingredients can produce synergistic effects, it has been considered that the crude extracts usually exert greater pharmacological activity than their active ingredients. In line with this notion, we obtained evidence that SCE has a broader pharmacologic effect than its active component SchB at the transcriptome level. These results provide insight into future research for developing therapeutic strategies. 

In summary, this study showed that SCE and SchB effectively reversed TGFβ-induced transcriptome changes in VSMCs. These results provide novel insight into future translational research for clinical application and for the development of healthcare strategies.

## Figures and Tables

**Table 1:** List of 38 MRs that control the expression of SCE-effective genes.

Entrez GeneID	Symbol	Description	FET *p*-Value ^1^	Markers in Regulon ^2^	Markers in Intersection Set ^3^	Mode ^4^
494322	Pms1	PMS1 homolog 1, mismatch repair system component	5.87 × 10^−37^	303	88	-
259241	Nr1d2	nuclear receptor subfamily 1, group D, member 2	1.51 × 10^−18^	387	73	-
83614	Pias3	protein inhibitor of activated STAT, 3	1.37 × 10^−17^	313	63	-
498945	Zfp90	zinc finger protein 90	5.01 × 10^−22^	237	60	-
499497	Mef2c	myocyte enhancer factor 2C	7.29 × 10^−20^	252	59	-
406164	Pbx2	PBX homeobox 2	3.22 × 10^−11^	315	52	-
308434	Zfp574	zinc finger protein 574	5.24 × 10^−23^	166	51	-
305972	Zfp395	zinc finger protein 395	3.13 × 10^−20^	168	48	-
83474	Tfam	transcription factor A, mitochondrial	1.70 × 10^−15^	154	40	-
690895	Zfp426	zinc finger protein 426	1.05 × 10^−14^	119	34	-
286979	Zfp455	zinc finger protein 455	1.74 × 10^−11^	143	33	-
308845	Thap12	THAP domain containing 12	4.10 × 10^−13^	112	31	-
309165	Rela	RELA proto-oncogene, NF-kB subunit	1.27 × 10^−8^	164	31	-
362667	Thap3	THAP domain containing 3	2.56 × 10^−7^	186	31	-
288489	Rbak	RB-associated KRAB zinc finger	5.57 × 10^−9^	150	30	-
85434	Cdc5l	cell division cycle 5-like	3.06 × 10^−14^	90	29	-
58851	Nr1h2	nuclear receptor subfamily 1, group H, member 2	8.15 × 10^−9^	127	27	-
293849	Zfp275	zinc finger protein 275	6.96 × 10^−9^	118	26	-
315689	Hmg20a	high mobility group 20A	2.01 × 10^−9^	104	25	-
58921	Foxm1	forkhead box M1	1.02 × 10^−8^	112	25	-
81785	Ssrp1	structure specific recognition protein 1	6.59 × 10^−16^	53	24	-
309452	Nfkb2	nuclear factor kappa B subunit 2	8.95 × 10^−10^	93	24	-
303518	Smarce1	SWI/SNF related, matrix associated, actin dependent regulator of chromatin, subfamily e, member 1	1.13 × 10^−6^	114	22	-
297758	Terf1	telomeric repeat binding factor 1	3.66 × 10^−6^	104	20	-
83514	Tsc22d3	TSC22 domain family, member 3	4.08 × 10^−5^	112	19	-
305827	Wdhd1	WD repeat and HMG-box DNA binding protein 1	1.36 × 10^−10^	48	18	-
361403	Terf2	telomeric repeat binding factor 2	7.48 × 10^−9^	47	16	-
313166	Nfx1	nuclear transcription factor, X-box binding 1	2.84 × 10^−8^	51	16	-
689883	Setdb1	SET domain, bifurcated 1	2.01 × 10^−7^	51	15	-
316580	Sp140	SP140 nuclear body protein	4.79 × 10^−6^	64	15	+
311604	Zhx3	zinc fingers and homeoboxes 3	2.61 × 10^−5^	73	15	-
303165	Zfp672	zinc finger protein 672	2.30 × 10^−5^	64	14	-
299810	Yeats4	YEATS domain containing 4	2.83 × 10^−6^	47	13	-
170849	Zfp111	zinc finger protein 111	9.62 × 10^−6^	52	13	-
315305	Atf1	activating transcription factor 1	1.20 × 10^−4^	65	13	-
117062	Hmga1	high mobility group AT-hook 1	3.29 × 10^−5^	50	12	-
312309	Zfp775	zinc finger protein 775	4.64 × 10^−6^	6	5	-
314616	Arid3a	AT-rich interaction domain 3A	1.83 × 10^−4^	6	4	+

^1^ The *p*-value from Fisher’s exact test. ^2^ The number of markers (genes) found to be first neighbors of the master regulator in the loaded network. ^3^ The number of markers found in the intersection of the signature and the regulon of the candidate MR. ^4^ The minus and plus signs respectively indicate down-regulation and up-regulation of master regulators.

**Table 2:** List of two MRs that control the expression of SchB-effective genes.

Entrez GeneID	Symbol	Description	FET *p*-Value	Markers in Regulon	Markers in Intersection Set	Mode
298400	Creb3	cAMP responsive element binding protein 3	1.02 × 10^−4^	44	5	-
360631	Mlx	MAX dimerization protein MLX	1.37 × 10^−4^	10	3	-

## Data Availability

The data presented in this study are available on request from the corresponding author.

## Supplementary Materials

The following are available online at https://www.mdpi.com/2075-1729/11/2/163/s1, Table S1: List of 1969 genes up-regulated by TGFβ versus vehicle; Table S2: List of 3552 genes down-regulated by TGFβ versus vehicle; Table S3: List of 2894 genes up-regulated by TGFβ + SCE versus TGFβ; Table S4: List of 994 genes down-regulated by TGFβ + SCE versus TGFβ; Table S5: List of 200 genes up-regulated by TGFβ + SchB versus TGFβ; Table S6: List of 651 genes down-regulated by TGFβ + SchB versus TGFβ; Table S7: List of 3999 genes up-regulated by SCE versus vehicle; Table S8: List of 2133 genes down-regulated by SCE versus vehicle; Table S9: List of 1275 genes up-regulated by SchB versus vehicle; Table S10: List of 1737 genes down-regulated by SchB versus vehicle; Table S11: List of 855 SCE-effective genes (red, up-regulation; blue, down-regulation); Table S12: List of 157 SchB-effective genes (red, up-regulation; blue, down-regulation); Table S13: List of TGFβ-induced upregulated Hallmark gene signatures that are reversed by SCE or SchB; Table S14: List of TGFβ-induced down-regulated Hallmark gene signatures that are reversed by SCE or SchB; Table S15: List of RelA target genes predicted by MRA.
